# To Operate or Not? Evaluating the Best Approach for First-Time Patellar Dislocations: A Review

**DOI:** 10.3390/jcm13185434

**Published:** 2024-09-13

**Authors:** Roberto Tedeschi, Daniela Platano, Federica Giorgi, Danilo Donati

**Affiliations:** 1 Department of Biomedical and Neuromotor Sciences, Alma Mater Studiorum, University of Bologna, 40136 Bologna, Italy; 2 Physical Medicine and Rehabilitation Unit, IRCCS Istituto Ortopedico Rizzoli, 40136 Bologna, Italy; 3 Pediatric Physical Medicine and Rehabilitation Unit, IRCCS Institute of Neurological Sciences, Sciences of Bologna, Via Altura 3, 40124 Bologna, Italy; 4 Physical Therapy and Rehabilitation Unit, Policlinico di Modena, 41125 Modena, Italy; 5 Clinical and Experimental Medicine PhD Program, University of Modena and Reggio Emilia, 41121 Modena, Italy

**Keywords:** patellar dislocation, conservative treatment, surgical treatment, knee function, redislocation rate

## Abstract

**Background:** The optimal management of first-time patellar dislocations remains a subject of debate. While surgical intervention is often recommended to reduce the risk of redislocation, the benefits over conservative treatment regarding knee function and complication rates are not clearly established. This systematic review aims to compare the effectiveness of surgical versus conservative treatment in preventing redislocation and improving knee function, while also considering complication rates. **Methods:** A comprehensive search of databases, including PubMed, Scopus, and Web of Science, was conducted up to July 2024. Seven randomized controlled trials involving 411 participants were included. The primary outcome was the redislocation rate, with secondary outcomes including knee function, complication rates, and the need for re-interventions. The quality of the studies was assessed using the PEDro scale. **Results:** Surgical treatment generally resulted in lower redislocation rates compared to conservative management. However, the improvement in knee function was inconsistent, with some studies showing no significant differences or even favoring conservative treatment. Surgical intervention was associated with a higher risk of complications and re-interventions, particularly in older studies with less advanced techniques. **Conclusions:** Surgical intervention appears effective in reducing redislocation rates but comes with a risk of complications. Conservative treatment remains a viable option, particularly for patients with lower activity levels or higher surgical risks. The decision between surgical and conservative management should be individualized, taking into account patient-specific factors and potential risks. Further high-quality research is needed to provide more definitive guidance.

## 1. Introduction

In the literature, acute patellar dislocation is a significant concern in orthopedic and sports medicine due to its prevalence, particularly among adolescents and females. This condition often leads to complications such as patellar instability, high redislocation rates, pain, challenges in returning to physical activities, and the potential development of patellofemoral osteoarthritis [1,2,3]. Despite its frequency, the optimal management approach—whether conservative or surgical—remains a subject of debate. Conservative treatment, which typically involves immobilization followed by rehabilitation, is favored for its non-invasive nature and potential to restore function without the risks associated with surgery. However, this approach has been criticized for its association with higher redislocation rates and insufficient prevention of long-term instability [4,5,6,7]. On the other hand, surgical intervention, which may involve techniques like medial patellofemoral ligament (MPFL) reconstruction, is advocated by some as it can directly address anatomical abnormalities and reduce the likelihood of redislocation. Nevertheless, surgery carries its own risks, such as infection, surgical complications, and longer recovery periods [7,8]. The current literature reflects this controversy, with studies yielding mixed results regarding the efficacy of both treatment modalities. Some randomized controlled trials (RCTs) suggest superior outcomes with surgical treatment in terms of reducing redislocation rates, while others indicate no significant difference in long-term knee function and quality of life between the two approaches [8,9,10,11]. Moreover, the methodological quality of these studies varies, with many suffering from limitations such as small sample sizes, lack of blinding, and short follow-up durations, which further complicates the ability to draw definitive conclusions. This systematic review aims to address this gap in the literature by rigorously evaluating the comparative effectiveness of conservative versus surgical treatment for acute patellar dislocation. By focusing on outcomes such as knee function, redislocation rate, quality of life, and return to sport, this review seeks to provide clearer guidance for clinicians managing patients with this condition. The ultimate goal is to inform evidence-based clinical decision-making and potentially contribute to the development of standardized treatment protocols.

## 2. Methods

The present review was conducted following the JBI methodology [12,13] for scoping reviews. The preferred reporting items for systematic reviews and meta-analyses extension for scoping reviews (PRISMA-ScR) [14,15] checklist for reporting was used.

### 2.1. Review Question

We formulated the following research question: “What is the comparative effectiveness of conservative versus surgical treatment in patients experiencing a first episode of acute patellar dislocation in terms of knee function, redislocation rate, quality of life, and return to physical activity and sport”?

### 2.2. Eligibility Criteria

Studies were eligible for inclusion if they met the following population, concept, and context (PCC) criteria.

Population (P): The population criteria included studies with participants of any age and gender who experienced a first episode of acute lateral patellar dislocation. These participants had no prior history of patellar instability, significant knee injuries, or underlying congenital or neuromuscular disorders that could affect the knee joint. The focus was on cases where the dislocation was primarily due to trauma or biomechanical factors.

Concept (C): The studies needed to evaluate and compare the outcomes of conservative versus surgical treatment approaches. Conservative treatments could include immobilization, physical therapy, and rehabilitation, while surgical interventions could involve procedures such as MPFL reconstruction or other realignment surgeries. The primary outcomes of interest were knee joint stability, redislocation rates, functional outcomes, quality of life, and return to sports.

Context (C): The studies had to be randomized controlled trials (RCTs) with a follow-up period of at least two years. The context included settings such as hospitals, clinics, or rehabilitation centers where both conservative and surgical treatments were administered and evaluated. Only studies published in English or Italian were included.

### 2.3. Exclusion Criteria

Studies that did not meet the specific PCC criteria were excluded.

### 2.4. Search Strategy

A comprehensive search strategy was employed to identify relevant studies for inclusion in this systematic review. The search was conducted across multiple databases, ensuring a thorough examination of the available literature. The databases searched, the specific search strings used, and the search period are detailed below.

#### 2.4.1. Databases Searched

PubMed;

Cochrane Library (CENTRAL);

PEDro (physiotherapy evidence database);

Scopus;

Web of Science.

#### 2.4.2. Search Period

We included manuscripts published between 2013 and 2024 in our search. The search was conducted from December 2023 to June 2024, with a final search conducted in July 2024 to ensure the most up-to-date studies were included.

#### 2.4.3. Search Strings

To capture all relevant studies, the search strategy utilized a combination of Medical Subject Headings (MeSH) and keywords related to the population, intervention, and outcomes. Boolean operators (AND, OR) were employed to effectively combine search terms.

PubMed Search String: (“Patellofemoral joint” [MeSH] OR “patellar dislocation” OR “patellar dislocation” [MeSH]) AND (“physical therapy” OR “conservative treatment” OR “rehabilitation” [MeSH] OR “exercise therapy” [MeSH] OR “treatment” OR “physical therapy modalities” [MeSH]) AND (trochleoplasty OR “orthopedic procedures” [MeSH] OR “surgery”).

#### 2.4.4. Cochrane Library Search String:

“Patellar dislocation” AND (“conservative treatment” OR rehabilitation OR “physical therapy” OR “physiotherapy”) AND (surgery OR trochleoplasty).

MeSH descriptors: [Patellar dislocation], [physical therapy modalities], [exercise movement techniques], [surgical procedures operative].

PEDro Search String: “Patellar dislocation” in abstract and title; Body part: lower leg or knee; Method: clinical trial; Match all search terms (AND).

Scopus and Web of Science: Similar search terms and Boolean logic were applied in Scopus and Web of Science, combining keywords and subject headings where appropriate to ensure a comprehensive search.

### 2.5. Study Selection Process

A total of 113 articles were identified through the database search. After removing duplicates, 69 articles remained, which were screened by reading the titles and abstracts. From this step, 56 articles were excluded for not meeting the inclusion criteria. The full text of the remaining 13 articles was assessed for eligibility, leading to the exclusion of 5 studies that did not meet the required population, concept, or context criteria. Finally, 8 studies were included in the systematic review.

#### 2.5.1. Additional Sources

No additional studies were identified through hand-searching reference lists or contacting authors, as full-text access was available.

This systematic search strategy was designed to ensure that all relevant randomized controlled trials comparing conservative and surgical treatments for acute lateral patellar dislocation were included in the review.

#### 2.5.2. Study Selection

The process described involves a systematic approach to selecting studies for a scoping review. Initially, search results were collected and refined using Zotero, with duplicates removed. The screening involved two levels: title and abstract review, followed by full-text assessment, both conducted independently by two authors with discrepancies resolved by a third. The selection adhered to the PRISMA 2020 guidelines [16], ensuring transparency and reliability. This rigorous methodology aimed to identify relevant articles that directly address the research question, maintaining a comprehensive and systematic approach in the review process.

### 2.6. Data Extraction and Data Synthesis

Data extraction for the scoping review was performed using a form based on the JBI tool, capturing crucial details like authorship, publication country and year, study design, patient characteristics, outcomes, interventions, procedures, and other relevant data. Descriptive analyses of these data were conducted, with results presented numerically to show the study distribution. The review process was clearly mapped for transparency, and data were summarized in tables for easy comparison and understanding of the studies’ key aspects and findings.

## 3. Results

**Figure 1:**
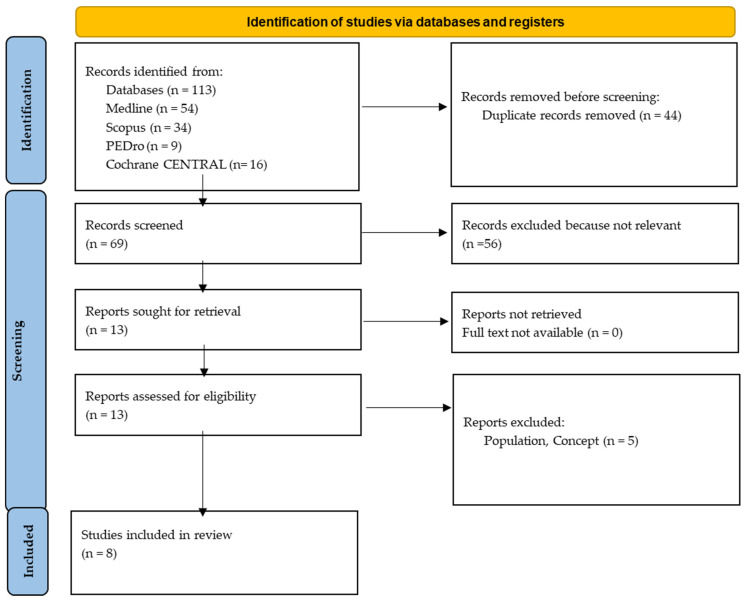
Preferred reporting items for systematic reviews and meta-analyses 2020 (PRISMA) flow-diagram.

**Figure 2:**
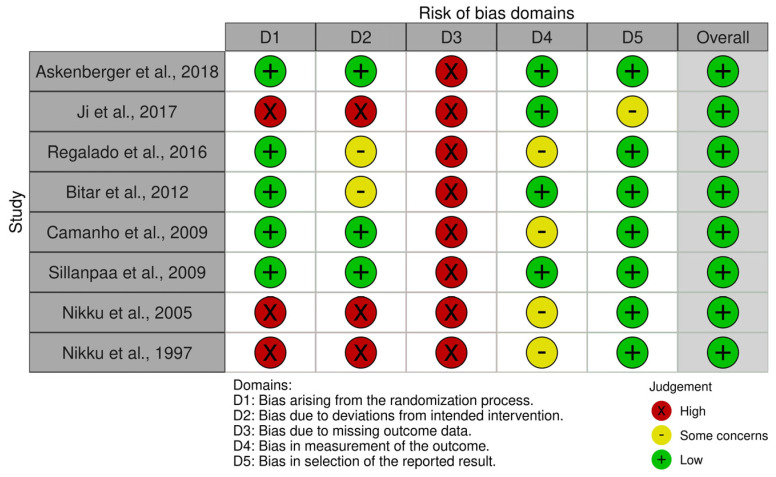
RoB–2 [4,7,17,18,19,20,21,22].

As presented in the PRISMA 2020-flow diagram (Figure 1), from 113 records identified by the initial literature searches, 105 articles were excluded and 8 were included (Table 1). The quality of the studies was assessed with the PEDro scale (Table 2) and RoB-2 scale (Figure 2), indicating the overall methodological rigor and risk of bias.

### 3.1. Redislocation Rate

Askenberger et al., 2018 [7], reported a statistically significant higher redislocation rate in the conservative group (16 redislocations) compared to the surgical group (8 redislocations) with an odds ratio of 2.76 (*p* = 0.047). Ji et al., 2016 [17], found no statistically significant difference in redislocation rates between the groups, though the surgical group had lower rates (3.3% redislocation, 6.7% subluxation) compared to the conservative group (11.5% redislocation, 15.4% subluxation). Regalado et al., 2016 [18], also reported a higher redislocation rate in the conservative group, with significant differences at both 3 and 6 years of follow-up (*p* = 0.03 and *p* = 0.02, respectively). Bitar et al., 2012 [4], found a 35% redislocation/subluxation rate in the conservative group, with no redislocations in the surgical group, a difference that was statistically significant. Camanho et al., 2009 [19], similarly reported no redislocations in the surgical group, compared to eight in the conservative group. Sillanpaa et al., 2009 [20], found a significant difference favoring the surgical group, with zero redislocations compared to six in the conservative group (*p* = 0.02). Nikku et al., 1997 [21], and Nikku et al., 2005 [22], both reported higher but not statistically significant redislocation rates in the conservative group, with younger age and specific anatomical factors predicting higher instability.

### 3.2. Knee Function (Kujala Score)

Ji et al., 2016 [17], reported a significantly higher Kujala score in the surgical group (*p* < 0.001). Bitar et al., 2012 [4], also found a higher Kujala score in the surgical group (*p* = 0.001), with a significant association between surgical treatment and better outcomes (OR 7.5; 95% CI, 1.9–30.0). In contrast, Askenberger et al., 2018 [7], found no significant difference in the Kujala score between groups. Camanho et al., 2009 [19], reported a higher Kujala score in the surgical group (mean scores: 92 for femoral condyle ligament insertion, 90 for patellar suturing) compared to the conservative group (mean score: 69). Sillanpaa et al., 2009 [20], found no significant difference in Kujala scores (*p* = 0.082) between the two groups. Nikku et al., 1997 [21], noted better objective function in the conservative group, although only specific physical tests showed significant differences. Nikku et al., 2005 [22], reported higher Kujala scores in the conservative group, with lower scores associated with female sex, higher Q angle, and the presence of loose bodies.

### 3.3. Subjective Function and Quality of Life

Askenberger et al., 2018 [7], found statistically significant differences in KOOS-child sports/play (*p* = 0.011) and KOOS-child QoL (*p* = 0.015), both lower in the surgical group. Ji et al., 2016 [17], noted that 80% of the surgical group rated outcomes as good/excellent, compared to 38.5% in the conservative group, though this difference was not statistically significant. Regalado et al., 2016 [18], reported similar knee function ratings between groups at 6 years, with a slight advantage for the surgical group. However, 4 patients in the conservative group and 2 in the surgical group were dissatisfied with their procedure. Bitar et al., 2012 [4], found a significantly higher percentage of good/excellent outcomes in the surgical group (*p* = 0.003). Sillanpaa et al., 2009 [20], reported no significant differences in subjective function scores between groups, although subjective assessments were better in the conservative group. Nikku et al., 1997 [21], reported better subjective function in the conservative group, with significant differences only in the Hughston VAS score (*p* = 0.04). Nikku et al., 2005 [22], found that lower subjective scores were associated with female sex, higher Q angle, and other anatomical factors.

### 3.4. Radiological Parameters

Ji et al., 2016 [17], reported statistically significant differences in patellar tilt (*p* < 0.001) and lateral shift (*p* = 0.001), with worse outcomes in the conservative group. Sillanpaa et al., 2009 [20], found no significant differences in radiological outcomes between groups, with one case of femoropatellar osteoarthritis in the surgical group. Nikku et al., 2005 [22], reported that an elevated Sulcus angle and lateral patellar displacement were predictive of the need for subsequent surgery.

### 3.5. Reoperations

Regalado et al., 2016 [18] reported no reoperations in the surgical group and nine reoperations in the conservative group at 3 and 6 years. Sillanpaa et al., 2009 [20], noted three reoperations in the conservative group and none in the surgical group (*p* = 0.24). Nikku et al., 1997 [21], and Nikku et al., 2005 [22], reported a higher number of reoperations in the surgical group, with younger age and specific anatomical factors being predictive of subsequent surgery.

## 4. Discussion

This systematic review aimed to evaluate the efficacy of surgical versus conservative treatment for first-time patellar dislocations by analyzing the redislocation rates, knee function outcomes, and complication rates across seven randomized controlled trials, encompassing a total of 411 participants. The inclusion of eight articles, where two reports by Nikku et al. [21,22] were considered as a single study due to overlapping cohorts, provided a comprehensive view of the current evidence on this topic.

The findings revealed a general trend favoring surgical intervention in reducing redislocation rates, yet the benefits on knee function were less clear, and the potential for postoperative complications remained a concern [4,23]. The primary outcome of the redislocation rate consistently showed lower rates in patients who underwent surgical intervention compared to those treated conservatively [15,24]. Six out of the seven trials demonstrated this trend, underscoring the effectiveness of surgical stabilization in preventing recurrent patellar dislocations. Notably, studies like Askenberger et al. (2018) [7] and Bitar et al. (2012) [4] reported statistically significant reductions in redislocation rates in their surgical cohorts.

However, it is important to acknowledge the limitations in these findings. The study by Nikku et al. (1997) [21] deviates from this pattern, as proximal realignment surgery failed to significantly lower the redislocation rate and was associated with a substantial number of complications. The findings from this older study reflect the evolution of surgical techniques, which have likely improved in precision and safety over the years. Moreover, the close proximity of the *p*-value to the significance threshold in some studies, such as the 0.047 *p*-value reported by Askenberger et al. [7] suggests that while surgery is generally more effective in preventing redislocations, the margin of superiority may not be as robust as it appears, necessitating careful interpretation.

Knee function, frequently assessed using the Kujala score, presented mixed results across the studies. While some, like Bitar et al. (2012) [4] and Ji et al. (2016) [17], showed a significant improvement in knee function post-surgery, others, such as Askenberger et al. (2018) [7] and Sillanpaa et al. (2009) [20], did not observe meaningful differences or even reported slight advantages for conservative treatment. This inconsistency likely stems from several factors, including the diversity of surgical techniques used, variations in rehabilitation protocols [25,26,27], and differences in patient demographics and baseline characteristics. The Kujala score, though widely used, may not fully capture the nuances of knee function, particularly in different population subsets.

Furthermore, a significant concern with surgical intervention is the risk of complications and the need for re-interventions. While surgery typically reduced redislocation rates, it also carried a higher burden of postoperative complications, especially in older studies like Nikku et al. (1997) [21] where severe outcomes such as sciatic nerve palsy and deep infections were reported. These complications emphasize the importance of surgical expertise and the selection of appropriate candidates for surgery. However, more recent studies, such as those by Sillanpaa et al. (2009) [20] and Regalado et al. (2016) [18], showed no re-interventions in their surgical cohorts, suggesting that when performed well, surgery can offer a durable solution with a low risk of long-term complications.

Additionally, the necessity for re-interventions, as reported in the Nikku et al. studies [20,21], where 61 additional procedures were required over seven years, serves as a cautionary tale about the long-term commitment and potential risks associated with surgical management. The review also brought to light the substantial heterogeneity in treatment protocols across the studies. Surgical techniques varied widely, from MPFL reconstruction and lateral release to more complex procedures like proximal realignment and trochleoplasty [28,29,30,31,32]. Such diversity reflects the tailored nature of surgical intervention, where the procedure is often chosen based on the patient’s specific anatomical abnormalities and the surgeon’s experience.

Moreover, emerging evidence highlights the importance of considering the differences in movement patterns between various patient types and sports. A study [33] emphasizes how different patient demographics, particularly those involved in sports, display varied biomechanical demands and compensatory movement patterns following knee injuries. These differences underscore the necessity of individualized rehabilitation approaches that account for the specific sports-related or daily activity requirements of the patient. Integrating these insights may further refine treatment protocols, potentially improving functional outcomes and reducing redislocation rates in specific subsets of patients.

This heterogeneity extends to rehabilitation protocols as well, with differences in the duration of brace use, timing of weight-bearing, and specific exercises recommended. These variations make it challenging to draw definitive conclusions about the superiority of one approach over another, as the outcomes are heavily influenced by the specifics of the intervention and the postoperative care provided. While surgical intervention generally reduces the risk of redislocation, personalized treatment approaches are essential. Factors such as age, activity level, and anatomical predispositions should guide treatment selection. For younger, more active patients, surgery may provide better outcomes, while conservative treatment could be more appropriate for those with lower physical demands or higher surgical risks.

### 4.1. Limitations

Several limitations were identified in the current body of evidence, which must be considered when interpreting the results of this review. The lack of blinding in most studies is a critical source of bias, as both participants and assessors were often aware of the treatment allocation, which could have influenced the reported outcomes. Additionally, the sample sizes in many trials were small, limiting the statistical power to detect significant differences between treatments. The absence of standardized outcome measures further complicates the synthesis of findings, as different studies prioritized different outcomes, making it difficult to compare results directly. The methodological quality of the included studies, as assessed by the PEDro scale, was generally low, with many studies failing to adequately describe randomization procedures or address potential confounders. These limitations suggest that the findings of this review should be interpreted with caution and highlight the need for more robust, high-quality trials in this area.

### 4.2. Implications for Clinical Practice

The results of this systematic review have several important implications for clinical practice. While surgical treatment appears to reduce the likelihood of redislocation, the decision to pursue surgery should be made on a case-by-case basis, considering the patient’s individual risk factors, activity level, and overall health. Conservative treatment remains a viable option, particularly for patients with lower physical demands or those at higher risk for surgical complications. Clinicians must balance the potential benefits of surgery, such as lower redislocation rates, with the risks of complications and the often unclear impact on long-term knee function. Patient education and informed consent are crucial, as patients need to be aware of the potential risks and benefits of each treatment option, allowing them to make informed decisions about their care. Patient selection plays a pivotal role in determining the success of both surgical and conservative treatments for first-time patellar dislocations. Factors such as age, physical activity levels, and specific anatomical characteristics (e.g., trochlear dysplasia, patella alta) should be carefully assessed. Younger, highly active patients with recurrent instability or athletes involved in high-impact sports may benefit more from surgical intervention, given their increased risk of redislocation. Conversely, older patients or those with lower activity levels may be better suited for conservative management, as the risk of redislocation is lower in these populations. Additionally, comorbidities such as obesity, generalized ligamentous laxity, and existing degenerative joint disease may tilt the balance toward conservative management or necessitate more specific surgical techniques. Emerging techniques in the management of patellar dislocations have the potential to significantly improve outcomes. For example, recent advances in minimally invasive MPFL reconstruction techniques, using smaller incisions and bioabsorbable anchors, have shown promise in reducing recovery time and minimizing complications. Additionally, innovations in rehabilitation protocols, such as neuromuscular training and the use of proprioceptive taping, have been proposed as adjuncts to both surgical and conservative treatments. Future research should focus on evaluating these novel approaches to better define their role in preventing redislocations and optimizing knee function.

## 5. Conclusions

This systematic review suggests that while surgical intervention generally reduces the risk of redislocation in first-time patellar dislocations, the decision to opt for surgery must be carefully weighed against the potential for complications and the often unclear benefits regarding knee function. Conservative treatment remains a viable alternative, particularly for patients with lower physical demands or when surgical risks are deemed too high. The findings highlight the need for individualized treatment plans, comprehensive patient education, and further high-quality research to establish more definitive guidelines for managing this condition. While surgical intervention appears to reduce redislocation rates, careful patient selection remains critical for achieving the best outcomes. Emerging surgical techniques and innovative rehabilitation strategies may further refine treatment protocols, helping clinicians to optimize care for individual patients.

## Figures and Tables

**Table 1:** Summary of included studies comparing surgical and conservative treatments for acute patellar dislocation.

Author, Year	Sample Characteristics and Inclusion/Exclusion Criteria	Surgical Treatment Group	Conservative Treatment Group	Follow-Up	Outcomes Measured
Askenberger et al. [7]	N = 74 (38 F, 36 M), age = 9–14 years. Stratification by sex. Block randomization of 6 patients. Patients aged 9–14 years with first acute lateral dislocation and hemarthrosis. Excluded prior knee injuries or lower limb disabilities. No osteochondral fragments > 1 cm² requiring open surgery.	37 patients, 29 analyzed at 2 years. Arthroscopic diagnosis of MPFL tear. Arthroscopic MPFL repair with TWINFIX Ti 3.5 QUICKT. Used 2–4 anchors depending on the lesion. Soft splint cast for 4 weeks with full weight bearing. Rehabilitation program identical to the conservative group.	37 patients, 29 analyzed at 2 years. Arthroscopic diagnosis of MPFL tear. Lateral stabilizing knee brace for 4 weeks with full weight bearing. Home exercise program and sessions with pediatric femoropatellar rehabilitation specialists. Strengthening exercises for thigh muscles, functional exercises, and gluteal and core strengthening.	Surgical group: Follow-up at 1 month and 3 months. Final follow-up at 2 years. Conservative group: Follow-up at 3 months. Final follow-up at 2 years.	Primary outcome: Redislocation rate. Postoperative complications, physical examination (apprehension test, thigh circumference 3 cm above patella, knee ROM, VAS), subjective function (KOOS-child, EQ-5D-Y QoL questionnaire, Kujala score, Tegner activity scale), objective function (1-legged hop test, side hop test, single limb 30 s mini-squat, and limb symmetry index (LSI)).
Ji et al. [17]	N = 62, 6 dropouts. Randomized by birth year. Patients with first dislocation within 3 weeks, without overlap-region injury. MPFL tear confirmed by MRI. Excluded prior knee injuries/surgeries and contralateral knee abnormalities.	30 patients. Open MPFL repair: tendon fixed distally to the condyle with a metal anchor and a suture. Brace in full extension with quadriceps strengthening. Knee flexion mobilization started after 2 days.	32 patients, 6 dropouts. Brace for at least 3 weeks with concurrent knee mobilization limited to 60° flexion, weight bearing as tolerated. Physiotherapy for 2–4 months until pain resolution and muscle strength recovery. Isometric quadriceps exercises, straight leg raises, global lower limb strengthening, with emphasis on VMO.	Mean follow-up of 42 months (24–54 months).	Primary outcome: Redislocation/subluxation rate. The apprehension test was used to assess patellar stability by subjective function (Kujala score, subjective questionnaire) and radiological parameters (patellar tilt and lateral shift on X-ray at 1 year post-treatment).
Regalado et al. [18]	N = 36, 6 dropouts at 6 years. Age = 8–16 years. 22 F, 14 M. Patients with first dislocation. Excluded prior knee injuries/surgeries, MPFL tear, or osteochondral fragments requiring surgery.	16 patients, 1 dropout. Lateral release (LLR) for 3 patients with isolated patellar instability. Modified Roux–Goldthwait procedure (proximal and distal realignment with LLR and medial imbrication) for 13 patients with patellar instability and/or tilt/misalignment. Brace and physiotherapy identical to the conservative group.	20 patients, 5 dropouts. Brace with lateral patellar support, allowing flexion up to 30° for 3 weeks, then up to 90° for the next 3 weeks. Physiotherapy for at least 3 weeks: Joint mobilization, isometric quadriceps strengthening, isokinetic exercises. Home exercise program. Brace removal at 6 weeks with full weight bearing from the start.	Follow-up at 3, 6, 12, and 24 months. Clinical and functional assessment at 36 months and functional assessment via telephone questionnaire at 72 months.	Primary outcome: Redislocation rate at 3 and 6 years. Reoperations at 3 and 6 years, postoperative complications, patient satisfaction with treatment, knee function questionnaire.
Bitar et al. [4]	N = 39, 41 knees: 20 F, 21 M. Randomized by lottery. Patients aged >12 years with first traumatic dislocation (no atraumatic dislocations) within 3 weeks. Excluded prior knee injuries/surgeries, MPFL tear, osteochondral fragments > 15 mm requiring surgery, and congenital or neuromuscular conditions.	21 patients, 21 knees, 3 dropouts. Mean age 23.95 years. Open MPFL reconstruction using the medial third of the patellar tendon fixed to the femoral epicondyle with a resorbable screw. Distal VMO border sutured to the new ligament. Full weight bearing after surgery. Brace for 3 weeks and physiotherapy identical to the conservative group.	18 patients, 20 knees, 2 dropouts. Mean age 24.10 years. Brace for 3 weeks in extension followed by physiotherapy including joint mobilization and isometric quadriceps strengthening, cryotherapy, and electrostimulation. Weight bearing allowed after 3 weeks. Cycling and proprioception exercises and closed and open kinetic chain exercises.	Mean follow-up: 44 months (24–61). Surgical group: Mean follow-up 38 months (24–48). Conservative group: Mean follow-up 48 months (24–61).	Primary outcome: Kujala score, redislocation/subluxation rate, presence of predisposing factors evaluated by X-ray (Crossing sign, trochlear bump, trochlear depth, patellar height), Kujala score related to group, sex, and affected knee, Kujala score related to age, and the relationship between predisposing factors and the Kujala score.
Camanho et al. [19]	N = 33, 20 F, 13 M. Randomized by lottery. Patients with first dislocation requiring reduction maneuver. Excluded ligamentous injuries or fractures requiring surgery and prior knee surgeries. Considered predisposing factors: Flat trochlea, valgus knee with angle >15°, high patella.	17 patients. Mean age 24.6 years. MRI to observe the lesion. MPFL repair. Arthroscopy for lesions near the patella. For lesions near the femur, the tendon was fixed to the epicondyle with anchors. Brace for 3 weeks, evaluated twice a week with flexion-extension exercises.	16 patients. Mean age 26.8 years. Groin-to-malleolar brace for at least 3 weeks, followed by 2–4 months of physiotherapy. Lower limb strengthening exercises, particularly for the VMO. Hamstring stretching started 1 month after trauma or surgery. Physiotherapy concluded after pain resolution and muscle strength recovery.	Mean follow-up of 40.4 months, patients visited every 6 months and asked about recurrences. Conservative group: Mean follow-up of 36.3 months.	Primary outcome: Redislocation or subluxation rate. The apprehension test was used to assess patellar stability based on the relationship between predisposing factors and redislocation in both groups and the Kujala score.
Sillanpaa et al. [20]	N = 40, 37 M, 3 F. Mean age 20 years. Randomized using sealed envelopes. Patients with first traumatic dislocation. Excluded prior dislocations or subluxations, other pathological conditions in either knee, osteochondral lesions requiring open surgery, or ligamentous injuries.	18 patients, 1 dropout. Technique chosen by 2 surgeons. Medial reefing for 14 patients (MPFL sutured with knee flexed at 30°), Roux–Goldthwait procedure for 4 patients. Brace and physiotherapy initiated 24–48 h post-surgery. All completed the first 6 weeks of aftercare and physiotherapy identical to the conservative group.	22 patients, 1 dropout. Patellar stabilizing brace. All completed the first 6 weeks of aftercare. Subsequent physiotherapy focused on lower limb strengthening. Full weight bearing allowed with extended knee for the first 3 weeks, ROM allowed between 0° and 30°. Isometric quadriceps strengthening exercises started initially. Brace removed at 6 weeks.	Mean follow-up of 7 years (6–9 years). Follow-up X-rays performed on all patients. Follow-up MRI performed on 29 patients.	Primary outcome: Redislocation rate, reoperations, return to previous activities, physical examination (ROM, VAS, thigh circumference 10 cm above patella), subjective function (Kujala score, Tegner activity scale), radiological parameters (Sulcus angle, lateral patellofemoral angle, patellar lateral displacement, degree of osteoarthritis), MRI to assess medial retinaculum or MPFL tears, and cartilage lesions.
Nikku et al. [21]	N = 125. Mean age = 20 years. Randomized by birth year. Patients with first lateral dislocation within 14 days. Excluded prior knee injuries or surgeries.	70 patients. Proximal realignment technique: Medial retinaculum repair via suturing, duplication, or additional reinforcement of the MPFL with adductor tendon in 63 patients. LLR in 54 of these patients. In total, 7 patients underwent LLR only. Brace and aftercare identical to the conservative group.	55 patients. Full weight bearing allowed, and strengthening exercises initiated as soon as possible. Patients with locked or dislocated patellae in EUA (regardless of group) were immobilized with the knee in neutral extension for 3 weeks, followed by mobilization in a patellar stabilizing brace for the next 3 weeks. Subluxated patients were mobilized with a brace for 6 weeks.	Mean follow-up of 25 months. Performance and provocation tests conducted on 123 patients, while the remaining 2 only completed the questionnaire.	Primary outcome: Redislocation rate, reoperations, postoperative complications, patient opinion via questionnaire, Tegner activity scale, Hughston VAS score, performance tests (10 min cycle ergometer, time for 3 laps on a figure-of-eight course, 1-leg hop test), provocation test (maximum number of squat downs in 1 min, then pain measurement (VAS)), and physical examination (thigh circumference 10 cm above patella, knee ROM, patellofemoral crepitus, apprehension test, scar, and peripatellar sensitivity).
Nikku et al. [22]	N = 125, 82 F, 45 M. Characteristics described in previous article.	70 patients, 40 aged < 16 years. Characteristics described in previous article.	55 patients, 30 aged < 16 years. Characteristics described in previous article.	Mean follow-up of 7 years.	Primary outcome: Redislocation/subluxation rate, reoperations, patient opinion via questionnaire, Kujala score, Tegner activity scale, and Hughston VAS score.

Legend. EUA: examination under anesthesia, KOOS: knee injury and osteoarthritis outcome score, LLR: lateral release, LSI: limb symmetry index, MPFL: medial patellofemoral ligament, MRI: magnetic resonance imaging, QoL: quality of life, ROM: range of motion, VAS: visual analog scale, VMO: vastus medialis obliquus.

**Table 2:** Quality assessment using the PEDro and RoB-2 scales (Figure 2), indicating the overall methodological rigor and risk of bias.

Author and Year	PEDro Score (Out of 11)
Askenberger et al. [7]	8
Ji et al. [17]	7
Regalado et al. [18]	6
Bitar et al. [4]	7
Camanho et al. [19]	6
Sillanpaa et al. [20]	7
Nikku et al. [21]	6
Nikku et al. [22]	6

Legend: PEDro score = physiotherapy evidence database score.
